# Perceptions about oncological physiotherapy among health and social care professionals and cancer care managers: a co-design approach for implementation strategies

**DOI:** 10.1007/s00520-025-09785-z

**Published:** 2025-08-18

**Authors:** Marta San Miguel-Pagola, Almudena Buesa-Estéllez, Pablo Gargallo-Aguarón, Patricia Roldán-Pérez, Marina Francín-Gallego, Pablo Bellosta-López, Lorena Villa-García, Almudena Medina-Rincón

**Affiliations:** 1 Universidad San Jorge, Campus Universitario, Autov. A23 Km 299, 50830 Villanueva de Gállego, Zaragoza, Spain; 2 Human Movement Sport Research Group, Faculty of Health Sciences, University of Zaragoza, Zaragoza, Spain; 3 REFiT Aging Research Group, Parc Sanitari Pere Virgili and Vall d’Hebron Institute of Research (VHIR), Carrer d’esteve Terradas, 30 Gracia 08023, Barcelona, Spain; 4 Department of Public Health, Mental Health and Mother-Infant Nursing, Faculty of Nursing, University of Barcelona, L’Hospitalet de Llobregat, Barcelona, Spain

**Keywords:** Co-design, World Café, Health and social care professionals, Cancer care managers, Oncological physiotherapy, Rehabilitation

## Abstract

**Purpose:**

This study aims to explore the knowledge and perceptions of health and social care professionals (HSCP) as well as cancer care managers and administrators (CCMA) in Spain regarding oncological physiotherapy. It seeks to identify barriers and propose strategies to enhance its integration into comprehensive cancer care.

**Methods:**

The World Café co-design methodology was employed to facilitate discussions among HSCP and CCMA. This approach, known for its dynamic, inclusive, and engaging nature, encouraged a wide range of perspectives and deeper insights through collaborative and adaptable conversations. The sessions were recorded, transcribed, and analyzed qualitatively using inductive thematic analysis.

**Results:**

Nineteen participants were involved, including 11 HSCP and 8 CCMA. The analysis revealed three primary themes: “Supportive Services,” “Physiotherapy Along the cancer continuum,” and “What Now?”. Key findings highlight the lack of awareness about the role of physiotherapy in oncology, significant barriers to its integration, and the need for more humanized healthcare. Participants emphasized the importance of interdisciplinary work, the inclusion of physiotherapy in all phases of the oncological process, and the role of case managers in coordinating care.

**Conclusions:**

These findings underscore significant gaps in the integration of physiotherapy into oncological care, including unmet needs due to lack of information, resources, and effective communication. Future efforts should focus on increasing the visibility of physiotherapy, integrating specialized physiotherapists into oncology teams, and enhancing the emotional education of professionals to provide more humanized care.

**Graphical Abstract:**

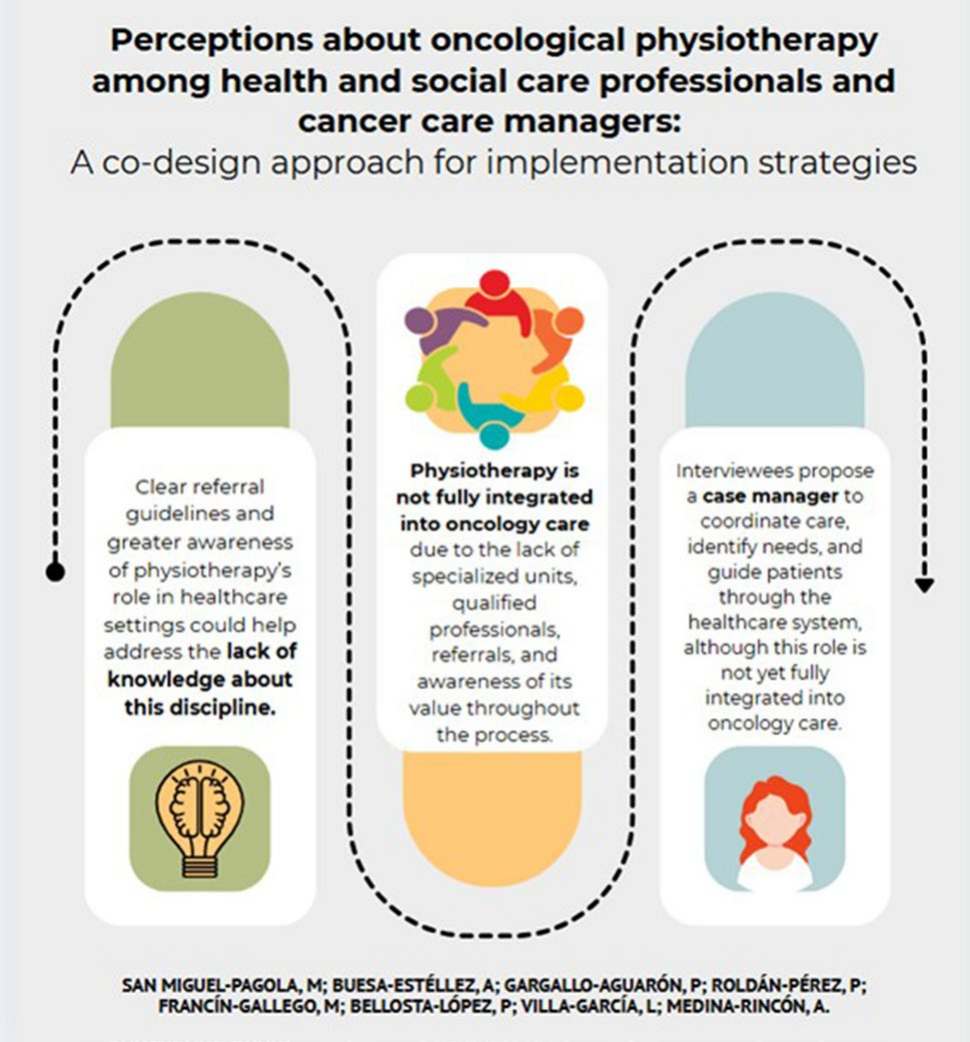

**Supplementary Information:**

The online version contains supplementary material available at 10.1007/s00520-025-09785-z.

## Introduction

Cancer remains a leading cause of morbidity and mortality worldwide, with its incidence steadily increasing [1]. In Spain, around 296,000 new cases are estimated for 2025, with projections reaching 350,000 by 2050. However, survival rates have significantly improved in recent years and are expected to continue increasing [2], largely due to advancements in prevention, early diagnosis, and new therapeutic options [1, 2].

Despite this, cancer survivors often suffer physical sequelae from the disease and its treatments impacting their functionality and quality of life, including fatigue, pain, lymphedema, musculoskeletal, cardiorespiratory, and neuropathies [3]. Recent studies indicate numerous unmet needs among cancer survivors, with physical challenges being most common [4]. This underscores the critical need for rehabilitation before, during, and after cancer treatment [5, 6].

Among rehabilitation professions, physiotherapy plays a crucial role in preventing and managing these sequelae. Numerous clinical guidelines recommend physiotherapy interventions [7, 8], and many studies propose its inclusion in interdisciplinary care teams to provide comprehensive support for people with cancer [6]. However, research has highlighted a discrepancy between clinical guideline recommendations and actual rehabilitation use in oncology, which remains underestimated and underutilized [6, 9].

Recognizing the magnitude of this issue, the World Health Organization has issued a call to action to improve access to rehabilitation for people with cancer [10]. Despite the well-documented benefits [7], physiotherapy is not routinely integrated into oncological care due to barriers including limited availability of physiotherapy services, legal and administrative restrictions on access, lack of financial coverage, absence of referral pathways, and a lack of awareness of its role in functional recovery [6, 11, 12]. This lack of knowledge is present among the general population [13], but also among health and social care professionals (HSCP) and Cancer Care Managers and Administrators (CCMA) linked to oncology [6, 11, 12].

This knowledge gap, combined with the need for accurate clinical guideline recommendations and standardized practices in oncological rehabilitation, can hinder referrals to physiotherapy services [6, 11]. Therefore, while understanding the knowledge level of patients and caregivers about oncological physiotherapy crucial for rehabilitation engagement [13], it is equally important to explore the knowledge of other stakeholders involved in facilitating access to these services. Higher knowledge among these stakeholders could facilitate the implementation of referral pathways to physiotherapy services and the integration of this discipline into standard cancer care [12, 14], ultimately addressing unmet rehabilitation needs.

Few studies have included professionals in coordination roles or oncology care management [15–18], with only one specifically addressing oncological physiotherapy [17]. Various qualitative methodologies have been employed to explore healthcare professionals’ perspectives on oncological rehabilitation [16–20], but none have used the World Café methodology. The World Café co-design methodology facilitates collaborative discussions and integrates diverse perspectives [13, 21, 22]. This inclusive approach fosters a deeper understanding of complex healthcare challenges by bringing together multiple stakeholders, thereby increasing the likelihood of successfully implementing new strategies [23, 24]. This study aimed to explore, using the co-design methodology, the perceptions and knowledge of HSCP and CCMA in Spain about oncological physiotherapy, and to understand the strategies they propose to promote its implementation in comprehensive care.

## Methods

### Co-design framework

This study employed the World Café method, a participatory qualitative approach that fosters collaborative knowledge creation through open discussions in an informal setting. It enables the exploration of experiences, identification of service needs, and co-design of solutions. The method combines rigor, relevance, and efficiency, making it particularly valuable for health service improvement [25, 26].

### Context and participants

#### Participant selection

The study was carried out at the Spanish Association Against Cancer (AECC) in Zaragoza, a non-profit organization of patients, relatives, volunteers, and oncology professionals active throughout Spain.

Eligible participants included health and social care professionals (HSCP) related to the oncological process and Cancer Care Managers and Administrators (CCMA). Exclusion criteria included acute physical or psychological conditions that could preclude meaningful participation or compromise the safety of the participant or others, as well as an expressed desire not to participate.

### Recruitment and sampling

In September 2023, participants were recruited by the AECC program psychologist and a research team member. They received an information sheet detailing the study objectives and provided written informed consent. The target was six to 12 participants per stakeholder group, with 13 invited to ensure sufficient participation [25]. A purposive, in-depth sampling approach was applied, considering a minimum of 1 year of work experience, representation from both public and private sectors, and diversity in age and sex to ensure heterogeneity. Given the context-specific nature of the study and the use of a captive group, an iterative approach to achieve theoretical saturation was not feasible. Instead, a pragmatic sampling strategy was adopted, guided by feasibility and the richness of the data generated within the available population [27].

### Data collection

#### Setting and data collection

Data was gathered through World Café sessions, a survey on participants’ sociodemographic variables, and researchers’ field notes. These sessions took place in person at AECC, in a quiet setting away from work areas to ensure privacy and avoid disruptions.

**Fig. 1:**
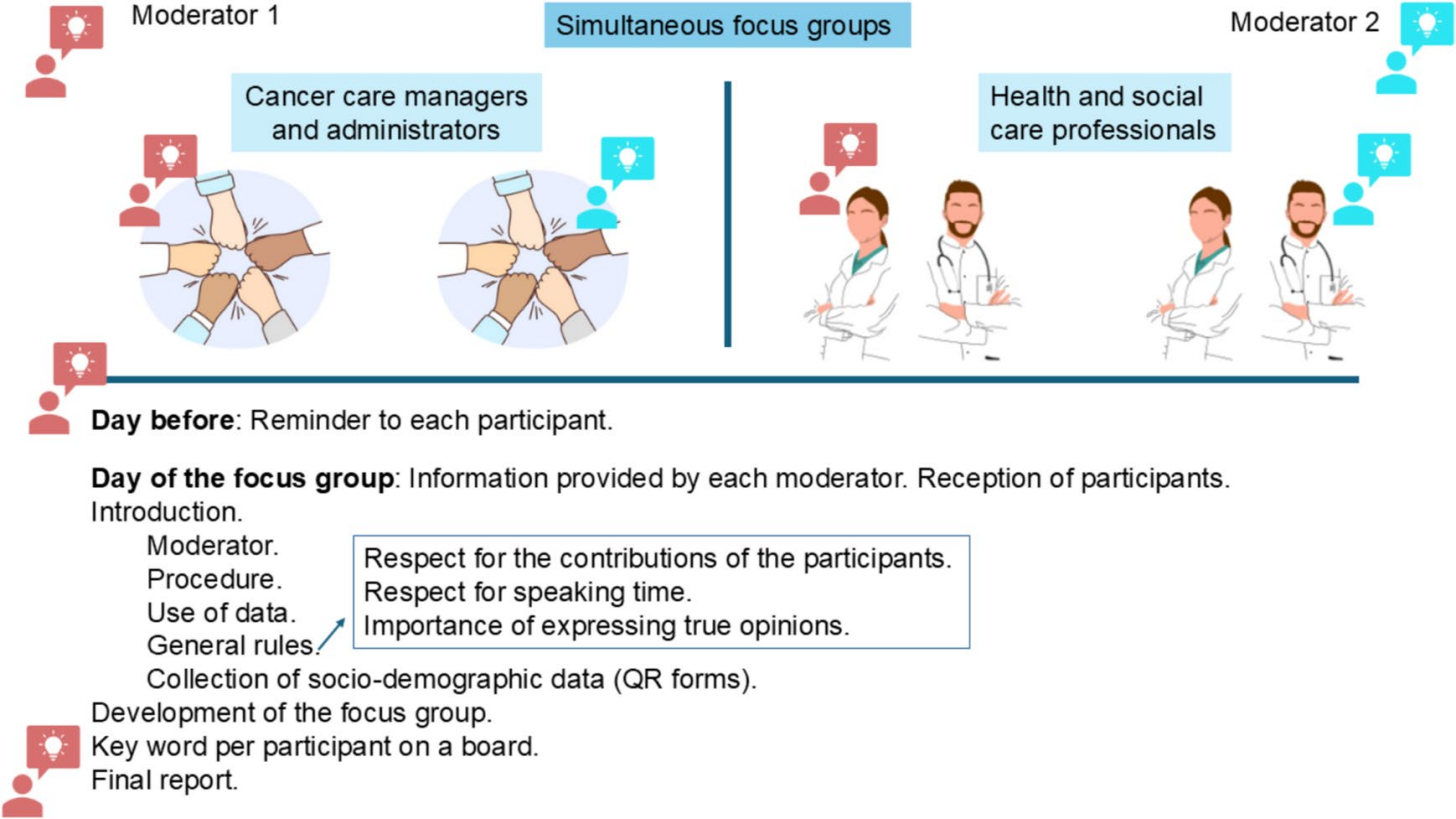
Procedure followed in the World Café sessions

Sessions were conducted between October and November 2023, following the procedure presented in Fig. 1. Each group session lasted ~ 2 h and consisted of a single round involving two simultaneous tables, each moderated by a facilitator. The moderators, who were part of the research team and experienced in cancer patient care and qualitative methodology, facilitated the sessions and played a central role as knowledge co-creators. They recorded systematic field notes in researchers’ notebooks to capture contextual details, non-verbal dynamics, and preliminary analytical insights throughout the process.

### Research questions

During the sessions, we followed a script of open-ended questions on categories of interest (supplementary material), developed from the research team’s experience review. The questions were previously tested with five individuals experienced in cancer rehabilitation to assess form, sequence, and content.

### Transcripts

Audio recordings were transcribed using the Amberscript software (Amberscript B.V., Amsterdam, Netherlands), ensuring fidelity to the original content without interpretation or modification. Participants’ identities were anonymized for confidentiality.

### Analysis

An inductive thematic analysis was conducted, following these steps: (i) reading transcriptions to identify initial themes and organize the data; (ii) identifying and classifying meaning units (MU), the smallest text segments, into codes or descriptive categories; (iii) grouping codes into units of analysis, common meaning groups (CMG), based on shared characteristics for further abstraction; and (iv) synthesizing content to create a narrative reflecting both content and meaning. Consensus on themes was reached after two triangulation sessions, with rigorous methodological strategies applied. The triangulation process was carried out among the research team members after the transcription and preliminary analysis of the results, during the second coding cycle.

### Methodological rigor

The Lincoln and Guba criteria were followed [28]. The Standards for Reporting Qualitative Research (SRQR) [29] and Consolidated criteria for reporting qualitative research (COREQ) standards ensured study validity [30]. Table 1 shows the techniques used to maintain rigor [31].

**Table 1:** Methodological rigor strategies

Criteria	Description	Technique used	
**Credibility**	Confidence in the “truth” of the findings	Research team triangulation	
		Member checking	
**Transferability**	Showing that the findings have applicablity in other contexts	Inspection of transcripts	
		Following a previously established correct protocol	
		Review the coding of specific parts	
		Communication between team members	
		Presentation of well-defined and described topics	
**Dependability**	Showing that the findings are consistent and could be repeated	Avoiding superficial coding	
		Avoiding the researcher’s interpretations	
		Adding negative cases	
		External auditor	
**Confirmability**	A degree of neutrality or the extent to which the findings of a study are shaped by the respondents and not researcher bias, motivation, or interest	Research herself/himself	Bracketing (researcher’s self-reflection on biases)
		Participants	Avoid social desirability bias
			Member checking
		Research team	Triangulation and Crystallization
			Peer debriefing
			Catalytic validity

### Ethical aspects

The study protocol adheres to the Helsinki Declaration and was approved by the Research Ethics Committee of the Autonomous Community of Aragón (CEICA), with the number: C.I. PI23/306. Data have been stored in line with General Data Protection Regulation guidance.

## Results

A total of 19 individuals participated, including 11 HSCP and 8 CCMA. The mean age was 49.9 [range: 32–72]. Among HSCPs, nine had over 5 years of cancer-related work experience, with seven employed in the Spanish public health system and six in private centers. Within the CCMA group, five participants were retired. Descriptive characteristics of all participant groups are presented in Table 2.

**Table 2:** Sociodemographic data of participants

Cancer care managers and administrators												
**ID**		**Gender**		**Age**		**Study levels**		**Employment situation**		**Years employed**		**Attributes**
A_01		Female		32		Postgraduate studies		Employed by a private company		1–5 years		Association coordinator and social worker
A_02		Female		56		University studies		Employed by a patient association		> 10 years		Association manager and history of breast and genital cancer
A_03		Female		61		University studies		Employed by a private company		Retired		Representative of a private entity and physiotherapist
A_04		Female		57		University studies		Retired		5–10 years		Association coordinator and history of metastatic breast cancer
A_05		Female		68		Completed secondary education		Retired		5–10 years		Association volunteer
A_06		Male		63		University studies		Retired		> 10 years		Association manager and physician
A_07		Female		64		University studies		Retired		> 10 years		Association coordinator
A_08		Male		72		Completed primary education		Retired		> 10 years		Association coordinator
**Health and social care professionals**												
**ID**	**Gender**		**Age**		**Study levels**		**Employment situation**			**Years employed**	**Characteristics**	
P_01	Male		33		University studies		Employed by a patient association		1–5 years		Association coordinator and social worker	
P_02	Female		45		University studies		Employed by the private healthcare system		> 10 years		Head of oncology service	
P_03	Female		36		Postgraduate studies		Employed by the public health system		> 10 years		Nurse and health school coordinator	
P_04	Female		39		Doctoral studies		Employed by the public health system		> 10 years		Breast cancer surgeon	
P_05	Female		37		Postgraduate studies		Employed by the public health system		1–5 years		Primary care physiotherapist	
P_06	Female		49		University studies		Employed by a patient association		> 10 years		Psycho-oncologist and association manager	
P_07	Female		32		University studies		Employed by a patient association		5–10 years		Social worker	
P_08	Female		54		Postgraduate studies		Employed by the public health system		> 10 years		Physiotherapist and supervisor of a physiotherapy unit	
P_09	Male		56		Postgraduate studies		Employed by the public health system		> 10 years		Oncologist	
P_10	Male		50		Doctoral studies		Employed by the public health system		> 10 years		Palliative care nurse	
P_11	Male		44		Doctoral studies		Employed by the public health system		> 10 years		Physiatrist	

### Descriptive results

**Fig. 2:**
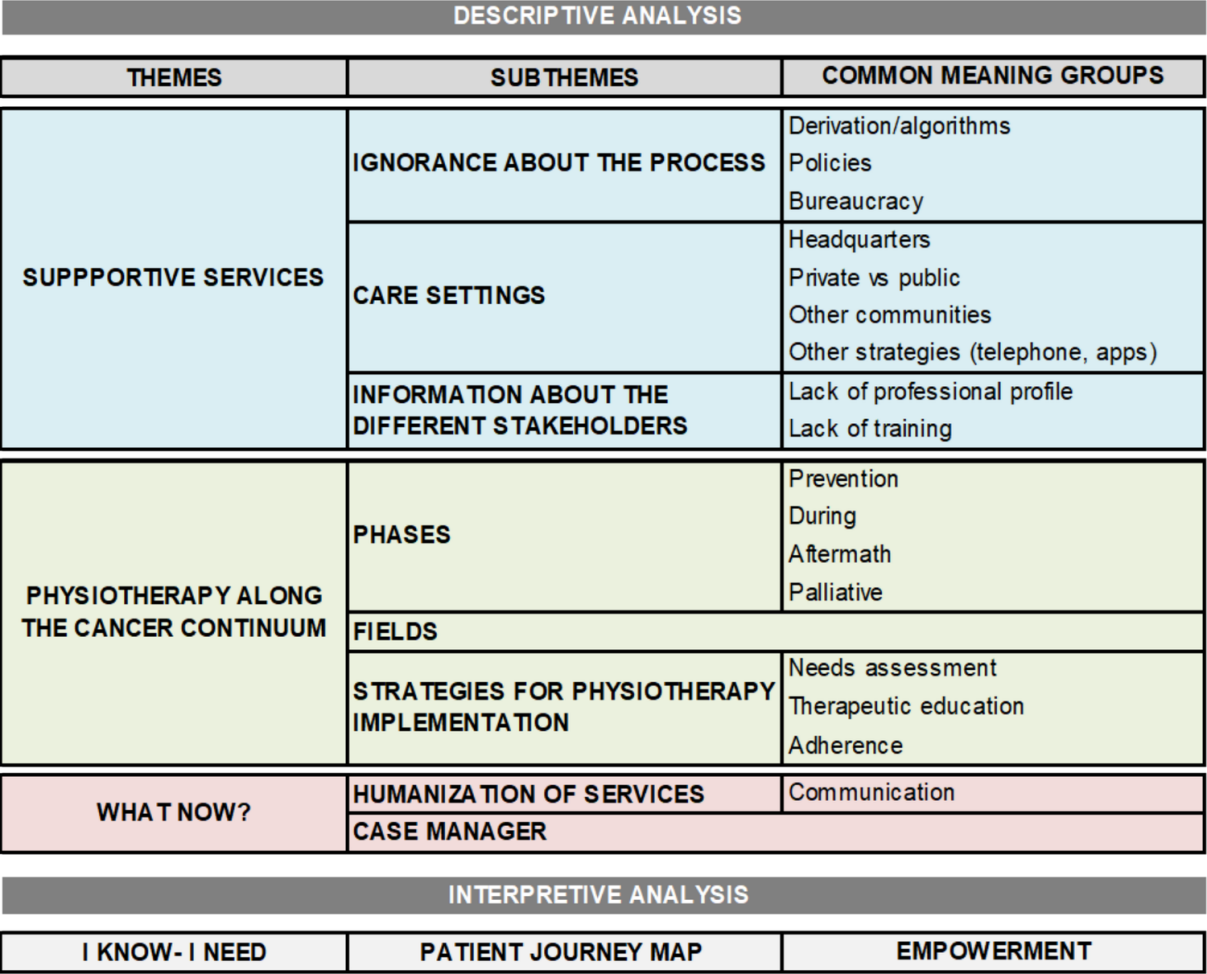
The path of an oncology patient

Three themes emerged from the inductive thematic analysis: “Supportive services,” “Physiotherapy along the cancer continuum,” and “What now?”. Figure 2 presents the organization of each sub-theme and its common meaning groups. Narratives illustrating each sub-theme and CMG are in the supplementary material.

#### Theme 1: Supportive services

Describe aspects related to cancer patient support services and the lack of awareness about access to them. These places of care, physically or not, depend on political decisions, public versus private health care, and referral algorithms. Many stakeholders orbit around the cancer patient, but each is often unaware of the others’ roles.

Subtheme 1.1. Ignorance about the process

Although physiotherapy in the oncological process exists, some professionals remain unaware of it.

“All health professionals should know that they can refer us to physiotherapy. The doctors themselves do not know that they can do it.” (P_05).

For cancer patients, accessing physiotherapy is often long and tedious, mired in bureaucracy, which increases waiting times.

“There are tremendous waiting lists. When the patient comes to me as a physiotherapist, and I tell him what is wrong with him, he tells me: I went for a consultation in August. And I say, my God, August, and we have him waiting in October. This cannot be.” (P_08).

The problem is not a lack of physiotherapists but rather a provision of resources, a decision made by politicians at the highest levels.

“There are no resources because there is no economic allocation. In other words, not because there is a lack of professionals, not because there is a lack of evidence, but because in the distribution of economic resources physiotherapy is not where it should be in the oncologic process.” (P_08).

Subtheme 1.2. Care settings

Cancer patients access physiotherapy services in hospitals or associations, whether publicly or privately managed, and use other strategies during their process.

The availability of an in-person place at the patients’ association is a predisposing factor for receiving physiotherapy.

“When the newly mastectomized patient comes, after the first diagnosis, apart from the fact that we visit them in the hospital when they are newly operated, they are received at the headquarters. Then we already talk to them about the need for physical activity and doing these kinds of things before the operation, during the treatment and after the treatment.” (A_02).

There is a significant difference in patient care between public healthcare and private services. Public health care often falls short due to limited time and the high number of patients.

“From my experience, I have been working for 30 years. The time we have in the physiotherapy services of the public system to treat the patient is very deficient to say the least.” (A_05); and access to private resources “Those who can afford it pay for it. If not, they are left with its aftermath.” (P_06).

Within the country, regional disparities in cancer patient care are evident.

“In other regions, physiotherapy does not play in a different league, it is a different sport.” (P_02).

“In rural areas access is more difficult.” (P_05).

As a result, alternative strategies for accessing physiotherapy, like telephone consultations, telerehabilitation, or finding professionals through associations, are sought.

“And neither he nor the patient must travel to the hospital, nor do you have to have people there who are taking up space. You’ve taught them and you're making sure that they’re adhering to the treatment.” (P_08).

Subtheme 1.3. Information about the different stakeholders

It is essential to understand and value the competencies of each member of the interdisciplinary team and the role of physiotherapy.

“Interdisciplinary work. We must know what each one of us does. Although efforts are made to include it, but there has been a time when society thought that physiotherapy are those of massage.” (P_11).

“That we all know what we all do and what really needs to be done, because that allows you to organize resources and to be clear that you are not wasting resources anywhere.” (P_08).

Proper training and continuous education for professionals are crucial, especially in communication and empathy.

“They have to be trained to be able to inform, otherwise it is impossible for them to know how to do it.” (P_07).

#### Theme 2: Physiotherapy along the cancer continuum

Once established, physiotherapy will be present throughout the oncological process, with its various specialties and through phases. The development of strategies to empower patients further highlights its importance.

Subtheme 2.1. Physiotherapy in different phases

Traditionally associated with managing sequelae, preventive physiotherapy is powerful and relevant even at the end of life.

“Physiotherapy before surgeries amazed me and it was a discovery for me because I did not know how important it could be.” (P_06).

“In the palliative patient, physiotherapy intervention improves, in addition to the symptoms, the patient’s dignity. Autonomy goes hand in hand with dignity.” (P_10).

Subtheme 2.2. Physiotherapy in different fields

Physiotherapy specialties can enhance the patient’s quality of life in various clinical aspects, including physical function for occupation and emotional well-being.

“Physiotherapy plays a fundamental role both in terms of health and work, because depending on that, I may or may not be able to work. Also emotionally, because it gives me a better quality of life. I think that in the end everything is like a puzzle, it has to fit together well.” (P_06).

Subtheme 2.3 Strategies for physiotherapy implementation

To enhance the value of physiotherapy, three key aspects can be seen as the different sides of a prism: “Needs assessment,” “Therapeutic education,” and active responsibility and “Adherence.”

“Patients should go from being inexperienced to experts.” (P_03).

“We have the population to be able to reply to us. We must share responsibility.” (P_02).

#### Theme 3: What now?

Professionals and association members agree on two key aspects: the need to humanize care, with communication as a basic competence, and the usefulness of the case manager role, ensuring each patient has a professional reference.

Subtheme 3.1. Humanization of services

Humanizing cancer patient care should mean a strong therapeutic alliance based on communication.

“They fill their mouths with humanization, and it is not putting new chairs and painting colored walls. Humanization is putting the person at the center and having an interdisciplinary team as we are talking about and considering all the aspects that make up a person’s life.” (P_01).

Subtheme 3.2. Case manager

Professionals demand the case manager role as a liaison between the social and healthcare environments, the patients, and their family. This person informs them of all available resources and necessary steps, ensuring continuous adaptation to their needs and coordinated care.

“A person of whatever category, but who is responsible for coordinating everything. Well, this patient has to go to psychology, he has to talk to the social worker, he has to go to physiotherapy. Someone of reference, whoever it is.” (P_11).

Interpretative results:

Based on the themes that have emerged, three cross-cutting ideas can be obtained highlighting physiotherapy in the oncological process:

1. *I know, so I need*. Being aware of all available options helps patients understand their needs better. Surgery, chemotherapy, radiation therapy, or immune-based therapies are accepted as necessary treatments. However, if patients are informed about all areas involved in the process, they can more accurately identify their needs and seek the appropriate referring professionals.
2. *Establish a patient journey map*. This powerful tool visualizes the patient’s journey from initial care to final discharge, providing clinicians with valuable insights into patients’ needs, emotional aspects, and opportunities for improvement, thus revolutionizing care delivery.
3. *Empowerment as a key*. Giving patients responsibility in decision-making, sharing common ground, valuing their experiences, and promoting adherence to self-care (e.g., therapeutic exercise) will change the disease experience.

## Discussion

To the best of our knowledge, this is the first study to explore the perspectives and level of knowledge of various HSCP and CCMA about oncological physiotherapy, using the World Café methodology. Participants perceive a limited presence and emphasize the need to expand physiotherapy in cancer care. Barriers to implementation mainly include a lack of awareness about the role and benefits of physiotherapy in all phases of the oncological process.

### Supportive services

This study reveals significant unawareness among HSCP and CCMA regarding the role of physiotherapy and rehabilitation in oncological care, a recurrent theme in the literature [6, 11, 17, 18]. A community professional noted the lack of awareness of physiotherapy’s relevance until patient demands highlighted its role. Some studies indicate physiotherapy as a highly demanded service by informed patients [19, 32–34]. Developing precise clinical referral guidelines or disseminating physiotherapy’s role within healthcare centers might be effective strategies to address this ignorance [6, 18, 35].

### Physiotherapy along the cancer continuum: prehabilitation, sequelae management, and palliative care

Testimonies reveal the critical role of physiotherapy in prehabilitation, helping patients undergoing surgery or treatments to improve or maintain physical function, promoting recovery, and reducing complications [8, 36]. Despite its benefits, barriers such as short periods between diagnosis and surgery and limited resources hinder implementation. In view of these challenges, participants emphasized prioritizing older adults or those with greater functional decline due to resource scarcity [37, 38].

Participants also discussed the role of physiotherapy in managing sequelae during or after treatments, such as lymphedema or pelvic floor dysfunctions. They emphasized physical exercise as a key intervention and the importance of referring patients with sequelae or at risk to physiotherapy units [7, 39]. However, they identified barriers such as scarce physiotherapy units, lack of referrals, and the need for specialized physiotherapists. Among the strategies proposed by some of them were to optimize resources and reach more patients, including group interventions [7, 36, 39] and digital platforms for exercise prescription and supervision [36, 40, 41].

Several reasons behind the lack of referrals to physiotherapy units were identified, including lack of awareness of the role and resources of oncological physiotherapy [17, 36], absence of these resources [15, 42], lack of standardized procedures [6, 11], poor care coordination [15, 17], and insufficient rehabilitation information provided to patients [35]. Additionally, some professionals still consider exercise prescription contraindicated in certain cases (e.g., with bone metastases or during treatment infusion), despite evidence of its safety and benefits. These barriers are documented in literature, which also proposes referral pathways based on patients’ needs and available resources [36, 39–41].

Physiotherapy also plays a crucial role in palliative care, enhancing quality of life and dignity. Participants emphasized its importance in advanced disease stages, aligning with previous research [17, 43].

### What now?

The lack of information provided to patients about available rehabilitation options is a common unmet need [4, 35]. Participants in our study highlight that patient organizations often fulfill this role within the healthcare system, advocating for their fundamental role in patient care to alleviate the system’s burden. Studies support the importance of community care in providing self-management educational tools and promoting adherence to rehabilitation treatment [19, 44]. However, professionals point to limited consultation time and excessive bureaucracy [11, 20, 36, 38], while inadequate resources in patient associations often drive patients to private physiotherapy, leading to access inequalities [10, 12, 45, 46].

Regarding the lack of coordination between care levels, respondents suggested creating a role to detect specific needs, refer patients to necessary services, and act as a liaison between different services. This case manager role, typically performed by nursing staff for chronic conditions [47], is not fully integrated into oncology care. Some studies suggest primary care physicians, nurses, or physiotherapists could fulfill this role in oncological rehabilitation [15, 18, 48].

The study participants emphasize the need to fully integrate physiotherapy and rehabilitation into comprehensive cancer care at different stages and include it as part of the interdisciplinary team, as suggested by other studies [12, 49]. They also urge administrations and legislators to invest more resources and highlight the need to expand knowledge about oncological physiotherapy and generate scientific evidence evaluating its cost-effectiveness, strategies previously proposed [15, 19, 42]. Additionally, they emphasize the need to strengthen competencies such as communication and empathy, enabling future professionals to provide person-centered and integrated care, thereby humanizing cancer care [50, 51]. These competencies must be integrated in a comprehensive framework involving technical knowledge about cancer rehabilitation [52].

### Strengths and limitations

Among the strengths of this study, we highlight the inclusion of a wide variety of participants, including health and social care professionals from different disciplines typically involved in the care of cancer patients. Additionally, the inclusion of managers and representatives from different sectors, such as public and private entities or patient associations for various oncological diseases, is noteworthy. Other strengths include conducting both descriptive and interpretative analyses and using the World Café methodology, which allows for greater freedom in participants’ responses and, therefore, the possibility of obtaining more comprehensive testimonies.

As limitations of this study, we could point out the lack of inclusion of primary care physicians and more administrative profiles with decision-making power in the implementation of physiotherapy services. Additionally, among health and social care professionals, the rural environment is not widely represented, which does not allow for an in-depth exploration of the situation of physiotherapy for cancer patients in this setting.

### Future directions

Future research could explore more advanced phases of co-design, including the implementation and evaluation of collaboratively proposed strategies with key stakeholders.

Additionally, it is imperative to conduct more research on the cost-effectiveness of implementing physiotherapy services in cancer care, as well as to develop access or referral pathways and standardized clinical practice processes so that oncological physiotherapy can benefit a greater number of patients. Furthermore, to understand the available resources, it would be interesting to create an observatory of oncological physiotherapy units in our country, involving national scientific societies. This could contribute to the transfer of successful models.

## Conclusions

Our findings suggest that physiotherapy is not fully integrated into the care of oncology patients due to the lack of specialized units and shortage of qualified professionals, the absence of referrals to existing units, and the lack of awareness among professionals and managers about the resources and potential of physiotherapy in all phases of the oncological process. To address these deficiencies, several solutions are proposed. Firstly, it is crucial to implement outreach strategies aimed at other healthcare professionals, social care providers, and cancer care managers. Additionally, it is essential to inform oncology patients about the importance of physiotherapy in the prevention and management of sequelae, as well as about the resources available both within the healthcare system and in the community. Cost-effective strategies such as education, self-management, the use of technologies, and group exercise interventions could also be promoted. Furthermore, continuous education and professional development are essential in both oncology-specific technical skills and crucial skills like communication and empathy. These elements are vital to adequately inform patients and provide high-quality care that supports them both technically and humanely throughout their cancer journey.

## Supplementary Information

Below is the link to the electronic supplementary material.

Supplementary file1 (DOCX 39 KB)

## Data Availability

The data underlying this study are available in the article and its online supplementary materials, except some words that have not been included due to the risk of re-identifcation.
