# Supplementary material for: Perceptions about oncological physiotherapy among health and social care professionals and cancer care managers: a co-design approach for implementation strategies
Source: Support Care Cancer. 2025 Aug 18;33(9):793. doi: 10.1007/s00520-025-09785-z (PMC12358330; doi:10.1007/s00520-025-09785-z)
Supplement: Supplementary file 1 — Supplementary file1 (DOCX 39 KB) [file 520_2025_9785_MOESM1_ESM.docx]

**Focus Group Question Guide**

| Shared questions across both focus groups | Perception of Physioherapy | What do you think physiotherapy can offer during the oncological process?  When do you think is the best time to receive physiotherapy treatment during the oncological process? Why? |
| --- | --- | --- |
| Focus group questions: cancer care managers and administrators | **Description of Experience with Physiotherapy Services** | Can you describe any previous experience with physiotherapy programs related to the oncological process?  Are you familiar with the oncological physiotherapy services available in the Aragón region? |
|  | *Accessibility* | How would you rate the accessibility of physiotherapy services/treatments for individuals diagnosed with cancer? Did the physiotherapists who treated you consider your needs, habits, and preferences? |
|  | *Environment* | Do you think there are differences between people diagnosed with cancer depending on whether they live in rural or urban areas of Aragón? If so, what are they? |
| Focus group questions: health and social care professionals | **Description of Experience with Physiotherapy Services** | How do you collaborate with physiotherapists involved in the treatment of individuals diagnosed with cancer?  Can you describe any previous experience with physiotherapy programs related to the oncological process? |
|  | *Referral Process* | How would you evaluate the experience of referring patients with oncological conditions to physiotherapy resources? |
|  | *Collaboration between physiotherapists and other professionals* | What are the strengths and weaknesses in collaboration with physiotherapists based on your experiences? |
|  | *Shared decision-making* | Do you believe physiotherapists are (or should be) involved in the decision-making process regarding treatment? |
|  | *Environment* | What differences do you observe between individuals with cancer living in rural versus urban areas? How does this influence your work? And the referral to physiotherapy services? |

**Remaining Focus Group Narratives**

| **THEME** | **SUBTHEME** | **COMMON MEANING GROUP** |
| --- | --- | --- |
| **SUPPORTIVE SERVICES** | **IGNORANCE ABOUT THE PROCESS** | **Derivation/algorithms**  “In lymphedema due to breast cancer. They do send you what you say once a year. In some hospitals, in others not." (A_02)  "But in this case, there are many doubts. Also, at first, if there is doubt that this has moved (metastasis), I have said that it does not touch me and if you touch someone, ask the oncologist. That must be ensured.” (A_02)  "I went to the oncologist who had the check-up six months later. I asked him; Hey, this radio frequency thing? He told me there is no problem, but nothing justification. Then I didn't come back either." (A_07)  "If not, she is going to be the one to sign. And then, of course, and then something happens." (A_02)  “Because it is that first gateway of the association in the hospital itself, in such a way that it is also aware of those needs that patients go through and that many times in the hospital.” (P_06)  **Policies**  “When I hear politicians talk about continuity, care, that the patient is at the center, that professionals have to accompany the patient.” (P_08)  “I mean, it's super sad, but until the importance and savings that physiotherapy can bring is demonstrated at an economic level, but until it is achieved. How much did the radio taxi service cost, which brought patients by ambulance and stuck all day for 15 minutes of radiotherapy? How much did you have to present at an economic level to decide to put taxis on them? Even if there are more taxis, it is cheaper.” (P_01)  “We are saving money, and we are contributing to the sustainability of the system, not only by accompanying the patient throughout their disease process, with which they have to be accompanied, but we are contributing to the sustainability of the entire health system.” (P_08)  **Bureaucracy**  “Bureaucracy and administrative management take up our time, we have to start not having to do so much administrative paperwork.” (P_03)  “It stresses me out infinitely more because I have a thousand papers to do, a thousand calls to patients, a thousand things that I have on my agenda.” (P_05)  “That time is decisive. But it is true that sometimes they could have the same five minutes and attend to me in a different way, because sometimes they just look or ask or what happens to you is that there are times when they stay like that waiting for you to tell them something. These are really basic communication skills.” (P_07) |
|  | **CARE SETTINGS** | **Headquarters**  "What happens is that the association is very scattered. So, as in Aragon, here we have nowhere to go or anything, we go virtual." (A_04)  "We when they come. In other words, in the first diagnosis, apart from visiting them in the hospital when they are recently operated, there are some who come at the first impact and are received at the headquarters. Then they are already told about the need for physical activity and doing this type of thing before the operation, during the treatment and after the treatment." (A_02)  "You have to federate and you have to register the association within the territory, if not, they don't give you an option or give you any help. " (A_07)  **Private Vs public**  "But this cost us a lot of time and a lot of money, because the psychologist at the Miguel Servet hospital paid more and attended to all the cancer patients every day. And so does physio." (A_02)  "So, what do you prefer, the mouth, the back in I don't know what, I don't know how much. Let's see where we go?" (P_08)  “Or what happens is that they manage themselves with money and they do it well. Of course, but because someone is asking for results, results, objectives. We have to start working.” (P_02)  “He pays it if he can. If not, it stays with their schools.” (P_06)  “Donations are a bit Game of Thrones. Because it is going one way, and it has to go that way. I can't go for the other one because the money comes through here and it can't be a donation.” (P_01)  **Other regions**  "But I tell you that in general older people, whether from towns or cities, are much more reluctant to associate." (A_07)  “It is necessary to make shared decisions, but a professional who helps from the beginning.” (P_02)  **Other strategies**  "I don't ask for that appointment anymore because for what? So that they measure my arm, since it is measured, measured by my physio and checked by me." (A_02)  **“**I will go to the next meeting of a manager of these I will go with well-defined criteria and I will say Criterion one: All cancer patients who are going to undergo surgery have to do prehabilitation to have fewer sequelae, fewer complications, less time in hospital for dad, Because this systematic review says so, Take the following criterion When they arrive at the ICU they need to do this, this, this if they are of this profile, I don't know what.” (P_08)  “With respect to technology, many times perhaps a rehabilitation exercise, he says. Jo, what a bummer Every day. But right now, there are technologies that are in all of them. Places less here, of course, attractive, entertaining, of course.” (P_06) |
|  | **INFORMATION ABOUT DIFFERENT STAKEHOLDERS** | **Lack of professional profile**  "It's that in public health. Incorporate it as part of cancer treatment." (A_07)  "Because that's what I also said that they do them the first 15 days, the 20." (A_04)  “Even of a multidisciplinary group sometimes really working on preparation from the point of view of physiotherapy or physical exercises.” (P_09)  “Physical exercise on behalf of the patient and directed. Physical exercise led by a professional, by a physio who says hey, because these exercise guidelines tone this, do that or even therapy.” (P_09)  “It is essential that oncology nursing, which is the one that is often in contact with the patient, especially in patients with intravenous treatments in the day hospital, even at the level of information, we would start with the consultation assistants who have that relationship with the patient.” (P_09)  “Perhaps it should be integrated, integrated into the oncological process in general, to get that chip that the breast part has.” (P_03)  “The professional must go down to earth.” (P_07)  “In my health center they have nursing sessions, pediatrics and medicine. Physio nothing.” (P_05)  “This even depends on what the hospital or outpatient service itself is like, that is, the health center. It's just that it's very variable. My oncologist told me I'm going to put you in the hands of a very good, excellent oncologist, but don't expect a word from him. Of course, then he saw me.” (A_06)  **Lack of training**  "A question: Is physiotherapy giving massages? ... I thought it was giving massages. Not just massages. Things about muscles." (A_04)  "When we basically use it (physiotherapy) in principle because of the problem of lymphedema. When you have surgery and your lymph nodes are removed, there's a problem in your arm.” (A_02)  "Manual techniques. It's a part of physiotherapy, which I have no idea about. There's more? Of course, of course." (A_04)  “Because at most now there is a boom in physiotherapy cabinets that are swarming everywhere. But what you make known is that I have put the window display, and I have put, I don't know that either.” (A_06)  "So now we have realized that we have a specific physiotherapist for this, for lymphatic drainage and for pressotherapy, and also to treat the problem, which is the same in the case of ovaries or genitals, which occur with lymphedema in the legs." (A_02)  “The will of the professional, and a little bit of the tact that you have now, a little bit of what you empathize with and what you want to dedicate.” (P_07)  “We have to understand, and we have to get to that because it is such a broad profession that I think yes, that we need to specialize, that you are specializing because of what you say already in postgraduate studies.” (A_03)  "And then, then there is the problem of pelvic floor rehabilitation, which is also very important in ovarian or uterine surgery or lower pelvic floor surgery, come on." (A_07)  "The Metastatic Breast Cancer Association wanted it to be discouraged. I don't understand why." (A_04)  "Exercise walking and doing things more or less gentle, gentle. I mean, like swimming, things seen. But they are afraid. For example, they have bone cancer. You can't." (A_04)  “My right leg falls asleep and it's because my lower back has also been affected for 12 years that you've been told not to move, not to take weight on this side and you've done everything with this one. Then this one has loaded.” (A_02)  "If they always pass, but they give you a piece of paper. A piece of paper with the exercises so that you can do things like this." (A_04)  “It is with the oncologist that they really have all the faith, let's say, of the treatment, the recovery and everything. So, the rest of the areas are a little lame. Everything else is like an accessory, let's say. So, I think it's that it's unknown, but it's also downplayed.” (P_07)  “Because I have had many patients who have not been told what lymphedema was and have had lymph nodes removed.” (P_03)  “We are very afraid to screw up. The patient is very afraid to do something that is not going to be done to him and so many times he does nothing so as not to screw it up by then. In that it is true that you need more information.” (P_09)  “We have subjects in our careers, but I think that in communication we fail.” (P_03)  “Many patients tell you that they haven't even looked at me. I was wondering as I looked at the computer.” (P_05)  “I know that you have the minimum knowledge of many things, and I can understand it, but no matter how much I understand it, it is not right.” (A_06) |
| **PHYSIOTHERAPY ALONG THE CANCER CONTINUUM** | **PHASES** | **Prevention**  “I, for example, go every 15 days or every 20 days. .... But I've been going for 12 years, and I started going for prevention very well. The problem is that it comes out at the same time because it doesn't come out on the same day, or you don't have to leave the same day of the operation. It came out after a year and a half, or two years and I started doing prevention.” (A_02)  "Do prevention things, which is the most important thing." (A_04)  "As far as I can speak in the ovary almost, the first step immediately after diagnosis is the operating room. I mean, that's in general. Other times they do interval surgery, but they are very countable cases." (A_07)  **During**  “We need to consider the benefits that physiotherapy provides for patients before, during, and after cancer treatment. Previously, it was often associated with very specific issues like lymphedema or general muscle pain from sports. However, the true importance of physiotherapy throughout the entire oncological process isn't always recognized.” (P_01)  "At the Hospital de Cartagena, they've set up a physiotherapy room specifically for oncology patients undergoing chemotherapy and radiation treatment. I saw videos of older women lifting weights, and it really helps them better tolerate the chemo. Things just feel better for them." (P_11)  **Aftermath**  "I believe that the problem is open. Because when you have surgery, this is practically annihilated. The main problem is the lack of mobility. So, in the association we are dedicated to this particular." (A_08)  “The exercises help me get the phlegm out. Respiratory therapy too. Yes. Yes." (A_08)  “Which limits, for example, at the level of personal suffering, inability to work and the problems that our patients give you with this type of sequelae.” (P_02)  “Sequelae that are approachable from physiotherapy and that would therefore lead to a better quality of life, better quality of life, better emotional well-being.” (P_08)  “I hear how important recovery, surgery or physical strength is during a treatment, The topic of muscle mass, having an active life.” (P_06)  **Palliative**  “We have to take a comprehensive approach and a comprehensive approach. Unfortunately, and as they unfortunately say in theory, you have to be a doctor, nurse, social worker and psychologist.” (P_10)  “The intervention of physiotherapy improves, especially something that we have talked about symptoms, but it improves the dignity of the patient, because there are more and more of them. But autonomy goes hand in hand with dignity.” (P_10)  “I believe that we have a significant shortage of training. Professionals in what is not, in the end-of-life process” (P_02) |
|  | **FIELDS** | “What about respiratory physiotherapy? And respiratory physiotherapy? 3/4 of the same. People associate more. And of course, manual therapy and exercise. Well, but, for example, respiratory physiotherapy. That is a great unknown. And eye, the impact it has on the patient's state of health.” (P_08)  “Physiotherapy, from my point of view, can accompany the oncology patient from the moment he/she is detected to prepare the body for what is to come and until the end of life.” (A_03)  “When we do the interconsultations, she is the one who, let's say, oversees distributing, classifying a little bit the treatments and of. And then to maintain a little bit, let's say, the order and flow of everything that is the reality of the oncologic process.” (P_11)  “We have to look for that autonomy. The sense of seeking autonomy is the one that I believe has to prevail in yours. And for that, physiotherapy is very useful, which I don't know.” (A_06) |
|  | **STRATEGIES FOR PHYSIOTHERAPY IMPLEMENTATION** | **Needs assessment**  “Lymphatic drainage treatment Manual for the prevention of lymphatic drainage Manual for the treatment Pressotherapy. What is the boot, this massage therapy for the cervical. The dorsal area and shoulders. Because if you don't use this, then you get this one. What's going on, we're here. I am with this and this area here is damaged both in the cervical and lumbar veins. ... And then we have the treatment that is also usual, not much, but quite a lot of the armpit cord. When you are operated on, it stays there like a cord because it is as if something stiff is left and then you cannot move this because it is hooked there. That also has to be dealt with and it has to be dealt with from the beginning. And we also have the treatment of the scar, the postoperative scar. And we make therapeutic exercise guidelines to recover, maintain shoulder mobility, prevent lymphedema, promote healthy lifestyle habits and sport. This is everything the physio does." (A_02)  “The relationship with physiotherapy because I had not seen it until the demands of the patients I attended began to demand this type of service.” (P_01)  **Therapeutic education**  “There has also been a change of mentality in the patient in terms of taking the patient, at least in my opinion, from having to be patient, he must be bedridden, he has to be standing. There has been a kind of change that is that the patient has discovered, or we have seen with evidence that moving the patient to stay active, to take physiotherapy treatment, rehabilitation produces a benefit.” (P_11)  “The patient feels less pain than you have explained. He faces the process he is going through in much better emotional conditions. It responds much better to when the physiotherapist goes. Then, as soon as he is operated on, that he has his surgery, his pain and that he is much more cooperative with the therapy because it has been explained to him before.” (P_08)  **Adherence**  "Then they know the mobility and the problems that patients can have, and they are delighted. The same people have been going for a year, the same people because they don't want to let go of the square." (A_02)  “How wrong it is for people not to take responsibility for their self-care, they have to really understand the benefit of exercise. They have to really understand, feel and sense the impact it has on themselves.” (P_08) |
| **WHAT NOW?** | **HUMANIZATION OF SERVICES** | “(After an emergency ovarian operation...) require more psychology services. In other words, the first thing. Psychology-Psycho-Oncology Services. Physiotherapy is a further step." (A_07)  "now it's there. Everything is very now with these years that they are dealing with humanization. So they give the information of the associations with the resources that they can give you the doctor. " (A_02)  “Maybe the leap would also have to be to expand home hospitalization, that the patient leaves the hospital much earlier, is cared for at home and culturally people would assume that you are in your house, but that the health professional comes and teaches you how to.” (P_08)  “And home hospitalization, health education that requires care, would enter all the patient knows Self-management in disease.” (P_11)  “And that the patient, obviously on many occasions, does grab it in the end, but many times he obviously looks in the mirror and says you are teasing me and the trust in the health care provider and in the family environment collapses.” (P_06) |
|  | **CASE MANAGER** | "Oncological processes do not address it in a comprehensive way. In other words, the doctor goes about his business, then the psychologist goes about his business." (A_07)  “A person who is of whatever category, but who is like the one responsible for coordinating, coordinating everything and who is why you say about launching. Well, look, this patient has to go to psychology, he has to talk to the worker, he has to go to physiotherapy, someone who is.” (P_11)  “There was a case management unit that was since it was a worker and a nurse and what they did is that every patient who was going to be discharged. First, they were going to make a visit with the nurse at the health center to see if there were architectural barriers.” (P_10) |
